# Rapid Antibiotic Susceptibility Testing of Tier-1 Agents *Bacillus anthracis*, *Yersinia pestis*, and *Francisella tularensis* Directly From Whole Blood Samples

**DOI:** 10.3389/fmicb.2021.664041

**Published:** 2021-07-09

**Authors:** Shahar Rotem, Ohad Shifman, Moshe Aftalion, David Gur, Tamar Aminov, Ronit Aloni-Grinstein

**Affiliations:** Department of Biochemistry and Molecular Genetics, Israel Institute for Biological Research, Ness-Ziona, Israel

**Keywords:** antibiotic susceptibility testing, whole blood, blood cultures, *Bacillus anthracis*, *Yersinia pestis*, *Francisella tularensis*, bloodstream infections, Tier-1

## Abstract

Rapid antibiotic susceptibility tests, performed directly on whole blood samples, will offer great clinical advantages. This issue is of considerable importance when it comes to bioterror pathogens where prompt antibiotic treatment should be offered to infected patients as well as prophylaxis to suspected exposed individuals. Herein, we describe a novel and rapid method, named MAPt, that is based on the direct application of a blood sample onto solid agar that has been embedded with different concentrations of the tested antibiotic. Following a short incubation, bacterial growth is monitored by qPCR. The method was applied on blood cultures and whole blood samples inoculated with the Tier-1 pathogens *Bacillus anthracis*, *Yersinia pestis*, and *Francisella tularensis*. The use of agar medium, which better supports the growth of bacteria at low concentrations, together with the use of qPCR, which provides sensitivity and specificity, allowed minimal inhibitory concentration (MIC) determination to a wide range of bacterial concentrations, ranging from ∼5 × 10^2^ cfu/ml up to 10^8^ cfu/ml. The omission of the enrichment procedure in blood culture and the isolation step, both required in standard antibiotic susceptibility tests (ASTs), allowed a dramatic reduction in time to answer, from a few days to a few hours. The total time required for MIC determination was ∼6 h for fast-growing bacteria, such as *B. anthracis*, and 12–16 h for slow-growing bacteria, represented by *Y. pestis* and *F. tularensis*. Accordingly, MAPt may offer health authorities means for public preparedness in the case of a bioterror attack as well as prompt clinical treatment options in common blood stream infections.

## Introduction

Bloodstream infections (BSIs) are a major public health concern (Goto and Al-Hasan, 2013). Rapid targeted antimicrobial therapy may reduce mortality and hospital length of stay. Special prompt care should be offered to BSIs originating from exposure to Tier-1 bioterror bacteria following a bioterror event or an endemic outbreak, as wide public health means of prophylactic treatment may also be needed. Unfortunately, these pathogens, as many others, may acquire antibiotic resistance either naturally due to extensive medical or agriculture use of antibiotics, or intentionally to improve their bioterror potential. Indeed, reduced susceptibility variants can be produced *in vitro* for all three agents: fluoroquinolone-resistant *Francisella tularensis* mutants may be obtained, some with cross-resistance to other clinically relevant antibiotic classes (Sutera et al., 2017), non-virulent isolates of *Yersinia pestis* with reduced susceptibility to ciprofloxacin or doxycycline were produced (Steinberger-Levy et al., 2016; Shifman et al., 2019), and *Bacillus anthracis* with reduced susceptibility to ciprofloxacin, ofloxacin and levofloxacin, moxifloxacin, garenoxacin, tetracycline, vancomycin, minocycline, quinupristin/dalfopristin, erythromycin, clindamycin, Penicillin G, and amoxicillin were generated by serial passage (Athamna et al., 2004). To note, clinical isolates of plasmid-mediated single and multiple drug-resistant strains have been isolated from plague patients (Guiyoule et al., 2001; Galimand et al., 2006). Bearing in mind the potential bioterror intensions implicated with these pathogens, there is a need for an antibiotic susceptibility test (AST) that will provide an adequate treatment option within a relevant clinical time frame. Upon hospital admission, cultures of three blood samples are taken from plague-suspected patients over 45 min before treatment (Gratz et al., 1999). Likewise, blood for culturing is taken from tularemia (Ellis et al., 2002; Tularemia Epidemiology, 2021) and anthrax-suspected patients (World Health Organization, 2008). These blood cultures may be used as a source for diagnostics and ASTs in those patients that do exhibit bacteremia during the course of the disease.

We have recently reported on a rapid AST, named MAPt (Micro-Agar-PCR-test), which allows one to address bioterror agent-contaminated environmental samples, offering rational targeted prophylactic treatment before the onset of morbidity in exposed individuals (Aloni-Grinstein et al., 2020). As MAPt does not require any enrichment, isolation, or quantification steps, and can be applied even at relatively low concentrations of bacteria, we evaluated its performance on clinical samples, blood cultures, and whole blood samples inoculated with three different Tier-1 agents: *B. anthracis*, *Y. pestis*, and *F. tularensis*. As most clinical ASTs require enrichment in blood cultures, which are grown for hours to days, following the isolation of the bacteria from the blood culture, implication of MAPt on clinical blood samples may offer great advantage time-wise as well as labor-wise.

In this study, we present the benefits of the use of MAPt on blood cultures and whole blood samples. We show that MAPt can be implemented on blood cultures and whole blood samples containing a broad range of bacterial concentrations, as low as 3.7 × 10^2^ cfu/ml and as high as 10^8^ cfu/ml. Thus, there is no need for the time-consuming blood culture enrichment step, which may take from a day to a few days. Moreover, blood components do not interfere with the procedure, omitting the need for an isolation/purification step of the tested bacteria. The results show that adequate MIC values are obtained for all three tested bacteria in significantly shorter time periods (hours) compared to the days required by the standard microdilution test. Thus, MAPt can be an attractive AST for bioterror agent-infected blood samples as well as for other clinical BSI infections.

## Materials and Methods

### Bacterial Strains, Media, and Growth Conditions

The *F. tularensis* live vaccine strain (LVS, ATCC 29684) was grown at 37°C on Cystine Heart Agar (5.1% CHA supplemented with 1% hemoglobin, Difco) or in cation-adjusted Mueller–Hinton broth (CAMHB; BBL, 212322), supplemented with 2% defined growth supplement (IsoVitaleX Enrichment; BBL 211876) and 3 μM hematin (Sigma 3281), termed HLMHI. The *Y. pestis* strain EV76 (Ben-Gurion and Shafferman, 1981), the spontaneous ciprofloxacin-resistant mutant #66-6 (Steinberger-Levy et al., 2016) and doxycycline-resistant mutant #36-4-18 (Shifman et al., 2019), and the *B. anthracis* strain Vollum ΔpXO1 ΔpXO2 (Levy et al., 2014) were grown on Brain Heart Infusion agar (BHI-A; BD Difco 241830) plates at 28°C and 37°C, respectively. Colony-forming unit (cfu) counts were determined by plating 100 μl of serial 10-fold dilutions in phosphate-buffered saline (PBS, Biological Industries, Beth Haemek, Israel) on CHA for *F. tularensis* and BHI-A plates for *B. anthracis* and *Y. pestis*.

### Blood Cultures and Whole Blood

Ten milliliters of human blood obtained from the National Blood Services, MDA, Israel, under MDA research permit 08-0122 was inoculated with different concentrations of *B. anthracis*, *Y. pestis*, and *F. tularensis*. The inoculated blood was transferred to BACTEC^TM^ Plus Aerobic/F Culture vials (BD, Cat# 442192). The blood cultures (10 ml blood) or whole blood (1 ml) inoculated with bacteria were shaken at 150 rpm at 37°C in a New Brunswick Scientific C76 water bath for various time periods.

### Preparation of MAPt Plates

The Micro-Agar-PCR-test plates were prepared using MHA (BD 225250) for *B. anthracis* and *Y. pestis* and CHA for *F. tularensis*. Agar media was prepared according to the manufacturer’s guidelines. Agar dilution was performed basically as described in CLSI standard M07 (CLSI, 2018). Following autoclaving, the agar was cooled to 50°C and 40 ml was aliquoted to 50-ml tubes where the tested antibiotic doxycycline (Sigma D9891), ciprofloxacin (ciproxin 200, Bayer), and gentamicin (Sigma G1264) were added. Antimicrobial solution (10×) was diluted by making twofold serial dilutions in master tubes. Then, one part of the 10× antimicrobial solution was added to nine parts of melted agar. Agar with no antibiotics served as growth control. One-hundred-fifty-microliter aliquots of the antibiotic-supplemented melted agar were divided into a 96-well plate.

### MAPt Assay

Ten microliters of the clinical sample (in duplicate) was plated in different wells of MAPt plates containing different concentrations of the tested antibiotics. The MAPt plates were incubated at the optimal growth temperature for each bacterial species (28°C for *Y. pestis* and 37°C for *B. anthracis* and *F. tularensis*) for the time required for each species. Of note, a 28°C incubation temperature for *Y. pestis* better supports bacterial growth without affecting the MIC (Frean et al., 2003; Hernandez et al., 2003; Heine et al., 2015), which led to shorter AST durations and easier MIC determination. Following the incubation period, the bacteria were extracted from the MAPt plates with 150 μl of PBS that was added to each well and pipetted up and down three times. One hundred microliters of the recovered bacteria was added to 100 μl of Triton buffer (20% Triton-X-100 in TE, Sigma), and the samples were heated for 30 min at 100°C in order to sterilize the sample and extract the DNA.

### qPCR Reactions

The qPCR reactions were performed in a total volume of 30 μl containing 2.3 μl of 20 mg/ml bovine serum albumin (BSA; Sigma A2153), 15.05 μl of SensiFAST Probe Lo-ROX Mix (Bioline BIO84005), 3.05 μl of forward primer (5 pmol/μl), 3.05 μl of reverse primer (5 pmol/μl), 1.55 μl of TaqMan probe (5 pmol/μl), and 5 μl of DNA extract. The primers and probes used were as follows:

For *B. anthracis*, the chromosomal marker targeting prophage lambdaBa03 (PL3; Weigel et al., 2010), PL3_F: AA AGCTACAAACTCTGAAATTTGTAAATTG, PL3_R: CAACG ATGATTGGAGATAGAGTATTCTTT, and Tqpro_PL3: FAM- AACAGTACGTTTCACTGGAGCAAAATCAA-BHQ-1. For *Y. pestis*, the gene *capR* encoding the Lon ATP-dependent serine protease (Steinberger-Levy et al., 2016), capF: GGATT ACGATCTCTCGGATGTGA, capR: AGCCGGACAGACGAAT AACTTC, and Taq-CapR: FAM-TTGTGGCGACCTCTAAC TCCATGAATATTCC-BHQ-1. For *F. tularensis*, the gene *fopA* encoding an outer membrane protein (Versage et al., 2003), fopAF: ATCTAGCAGGTCAAGCAACAGGT, fopAR: GTCAACACTTGCTTGAACATTTCTAGATA, and fopAP: FA M-CAAACTTAAGACCACCACCCACATCCCAA-BHQ-1. The PCR thermal conditions were as follows: 3 min at 60°C followed by 40 cycles of 15 s at 95°C and 35 s at 60°C.

### Quantification of Bacterial Growth Inhibition by qPCR

Bacterial quantification by qPCR was determined using the Ct value, which was extracted by the 7,500 real-time PCR system Sequence Detection Software (version 1.4). The relative difference in bacterial growth between an untreated control and an antibiotic-treated sample (designated FC) was calculated by the formula FC = 2^–ΔCt^, where ΔCt is the difference between the Ct of sampled bacteria compared to the Ct of the untreated control sample. A 10-fold change between the antibiotic-treated and untreated samples is reflected by ΔCt = 3.3 (log_2_10) in an efficient PCR; thus, the MIC was defined as the lowest antibiotic concentration that reduced growth to ΔCt ≥ 3.3, which correlated with the lack of visible growth by the standard AST.

### MIC Determination by Broth Microdilution

Standard broth microdilution was performed according to the CLSI guidelines (Clinical and Laboratory Standards Institute, 2010) in CAMHB for *B. anthracis* and *Y. pestis*, and HLMHI for *F. tularensis*. An inoculum of 5 × 10^5^ to 1 × 10^6^ cfu/ml suspended in CAMHB for *B. anthracis* and *Y. pestis*, and 2 × 10^6^ cfu/ml for *F. tularensis* were added at a 1:1 volumetric ratio to a 96-well plate (TPP, Cat# 92696) containing duplicates of twofold serial dilutions of doxycycline (Sigma D9891) or ciprofloxacin (ciproxin 200, Bayer) in CAMHB or HLMHI at a final volume of 0.1 ml. Bacteria grown in CAMHB or HLMHI without the addition of doxycycline or ciprofloxacin served as growth controls in each assay. The 96-well plate was incubated at 37°C for 20 h for *B. anthracis*, 28°C for 24 h for *Y. pestis*, and 37°C for 48 h for *F. tularensis* in an Infinite 200 plate reader (TECAN), and growth was monitored by measuring the optical density at 630 nm (OD_630_) at 1-h intervals. A 28°C incubation temperature for *Y. pestis* was used because it better supported bacterial growth without affecting the MIC (Frean et al., 2003; Hernandez et al., 2003; Heine et al., 2015), which led to shorter AST durations and easier MIC determination. The MIC values were defined as the lowest doxycycline and ciprofloxacin concentrations that reduced growth to less than 10% of the OD_630_ measured for the growth control. No growth was verified by visual inspection.

## Results

### MIC Determination by MAPt to Blood Cultures and Whole Blood Samples Inoculated With *Francisella tularensis*

**FIGURE 1:**
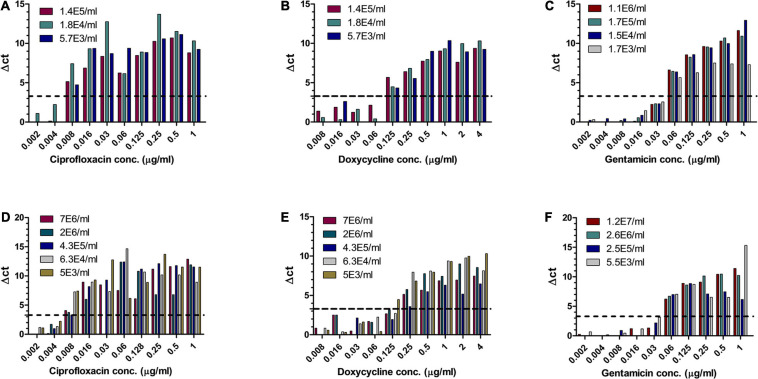
MIC determination of blood cultures and whole blood samples spiked with *F. tularensis*. Human blood cultures **(A–C)** and human whole blood samples **(D–F)** inoculated with different concentrations of *F. tularensis* (cfu/ml) were subjected to AST for ciprofloxacin, doxycycline, and gentamicin by the MAPt analysis. MICs were determined, as described in the *Materials and Methods* section, by the threshold ΔCT = 3.3.

Of the three Tier-1 agents, *F. tularensis* is the most challenging to determine antibiotic susceptibility in terms of time. By the standard AST method, at least 5 days are required to determine antibiotic susceptibility. Dependent on the initial bacterial concentration, 1–4 days are required for a blood culture to flag positive (Shifman et al., 2021), 2 days are required for isolation, and 2 days are required for the standard microdilution test. To examine the applicability of MAPt, several concentrations of *F. tularensis* were inoculated into human blood cultures and allowed to grow for various lengths of time, yielding a range of concentrations of *F. tularensis* blood cultures. Similarly, whole blood samples inoculated with various *F. tularensis* concentrations were grown for 2 h. All samples were applied to MAPt plates containing ciprofloxacin, doxycycline, or gentamicin, antibiotics that are recommended treatments for *F. tularensis* (CDC, 2018), followed by incubation for 14 h at 37°C and qPCR to determine growth in the presence of the tested antibiotics. MIC values obtained by MAPt within 16 h, for all bacterial concentration tested, are presented in Tables 1, 2, and agree with those obtained by the standard microdilution test (ciprofloxacin 0.008–0.016 μg/ml, doxycycline 0.125–0.25 μg/ml, and gentamicin 0.032–0.06 μg/ml), which requires a defined bacterial concentration of 2 × 10^6^ cfu/ml. Graphic representations of the ΔCT results, for representative blood cultures and whole blood samples, for all three tested antibiotics, are depicted in Figure 1, showing the 10-fold change between the antibiotic-treated and the untreated sample as reflected by ΔCt = 3.3 (log_2_10).

**TABLE 1:** MIC values for ciprofloxacin and doxycycline obtained by MAPt of blood cultures and whole blood samples inoculated with *F. tularensis*.

**Sample (cfu/ml)**	MIC (μg/ml) by MAPt*	
	**Ciprofloxacin**	**Doxycycline**
**Blood cultures**		
4.5 × 10^3^	0.008	0.125
5.7 × 10^3^	0.008	0.125
1.5 × 10^4^	0.008	0.125
1.8 × 10^4^	0.008	0.125
2.4 × 10^4^	0.016	0.125
1.4 × 10^5^	0.008	0.125
**Whole blood**		
5 × 10^3^	0.016	0.125
6.3 × 10^4^	0.008	0.25
4.3 × 10^5^	0.008	0.25
2 × 10^6^	0.008	0.25
7 × 10^6^	0.008	0.25
MIC by standard AST**		
2 × 10^6^	0.008–0.016	0.125–0.25

Duration of the test: *MAPt: 16 h. **Standard test: 4–5 days.

**TABLE 2:** MIC values for gentamicin obtained by MAPt of blood cultures and whole blood samples inoculated with *F. tularensis*.

**Sample (cfu/ml)**	MIC (μg/ml) by MAPt*
	**Gentamicin**
**Blood cultures**	
1.7 × 10^3^	0.06
4.4 × 10^3^	0.06
1.3 × 10^4^	0.06
1.5 × 10^4^	0.06
1.2 × 10^5^	0.06
1.7 × 10^5^	0.06
10^6^	0.06
1.1 × 10^6^	0.06
**Whole blood**	
4 × 10^3^	0.06
5.5 × 10^3^	0.06
4.1 × 10^4^	0.03
2.5 × 10^5^	0.06
4.6 × 10^5^	0.06
2.6 × 10^6^	0.06
3.9 × 10^6^	0.06
1.2 × 10^7^	0.06
MIC by standard AST**	
2 × 10^6^	0.032–0.06

Duration of the test: *MAPt: 16 h. **Standard test: 4–5 days.

### MIC Determination by MAPt to Blood Cultures and Whole Blood Samples Inoculated With *Yersinia pestis*

**FIGURE 2:**
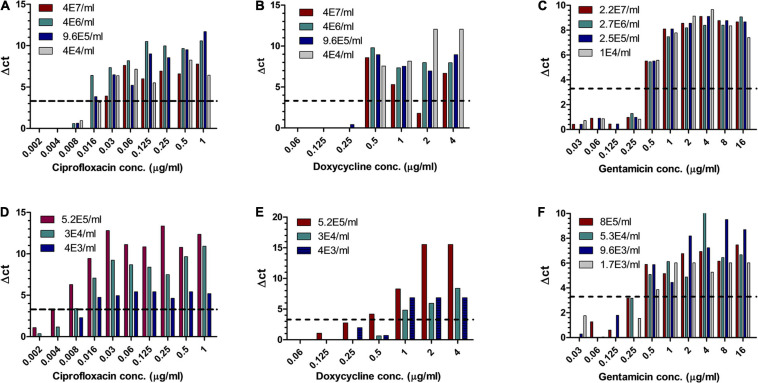
MIC determination of blood cultures and whole blood samples spiked with *Y. pestis*. Human blood cultures **(A–C)** and human whole blood samples **(D–F)** inoculated with different concentrations of *Y. pestis* (cfu/ml) were subjected to AST for ciprofloxacin, doxycycline, and gentamicin by the MAPt analysis. MICs were determined, as described in the *Materials and Methods* section, by the threshold ΔCT = 3.3.

Of the three Tier-1 agents, *Y. pestis* is clinically the most challenging one as *in vitro*, its growth rates are slow, but *in vivo*, the bacteria proliferate quickly, leading to severe disease and death within 24 h following symptom onset (Inglesby et al., 2000), at times before AST results can be obtained. Hence, the ability to determine antibiotic susceptibility before deterioration bears clinical value. Similar to the method described for *F. tularensis*, different concentrations of *Y. pestis* were inoculated into human blood cultures and whole blood samples and allowed to grow for various lengths of time, yielding a range of bacterial concentrations in the samples. The samples were then applied to MAPt plates, incubated for 12 h at 28°C and subjected to qPCR to determine growth in the presence of the tested antibiotics. Tables 3, 4 summarize the results obtained by MAPt, showing suitable MIC values compared to the standard microdilution test (ciprofloxacin 0.008–0.032 μg/ml, doxycycline 0.5–1 μg/ml, and gentamicin 0.25–1 μg/ml), which requires a defined bacterial concentration and an overall time of 2–3 days. Graphic representations of the ΔCT results for blood cultures and whole blood are illustrated in Figure 2.

**TABLE 3:** MIC values obtained by MAPt of blood cultures and whole blood samples inoculated with *Y. pestis*.

**Sample (cfu/ml)**	MIC (μg/ml) by MAPt*	
	**Ciprofloxacin**	**Doxycycline**
**Blood cultures**		
4 × 10^4^	0.032	0.5
9.6 × 10^5^	0.016	0.5
4 × 10^6^	0.016	0.5
4 × 10^7^	0.032	0.5
**Whole blood**		
4 × 10^3^	0.016	1
1.6 × 10^4^	0.016	0.5
3 × 10^4^	0.016	1
1.4 × 10^5^	0.016	1
5.2 × 10^5^	0.008	0.5
1.3 × 10^6^	0.016	0.5
1.4 × 10^8^	0.008	1
MIC by standard AST**		
5 × 10^5^	0.008–0.032	0.5–1

Duration of the test: *MAPt: 12 h. **Standard test: 2–3 days.

**TABLE 4:** MIC values for gentamicin obtained by MAPt of blood cultures and whole blood samples inoculated with *Y. pestis*.

**Sample (cfu/ml)**	MIC (μg/ml) by MAPt*
	**Gentamicin**
**Blood cultures**	
10^4^	0.5
2.5 × 10^5^	0.5
2.7 × 10^6^	0.5
2.2 × 10^7^	0.5
**Whole blood**	
1.7 × 10^3^	1
2.4 × 10^3^	0.5
9.6 × 10^3^	0.5
1.4 × 10^4^	0.5
5.3 × 10^4^	0.5
1.6 × 10^5^	0.5
8 × 10^5^	0.5
1.4 × 10^6^	0.5
MIC by standard AST**	
5 × 10^5^	0.25–1

Duration of the test: *MAPt: 12 h. **Standard test: 2–3 days.

### MIC Determination by MAPt to Whole Blood Samples Inoculated With *Bacillus anthracis*

**FIGURE 3:**
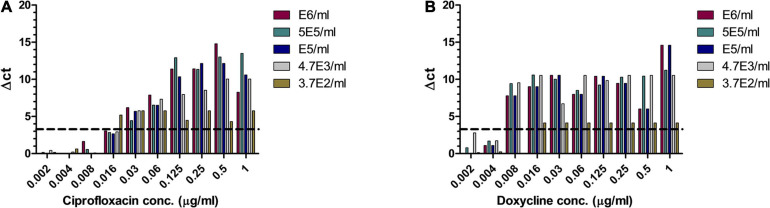
MIC determination of whole blood samples spiked with *B. anthracis*. Human whole blood samples inoculated with different concentrations of *B. anthracis* (cfu/ml) were subjected to AST for ciprofloxacin **(A)** and doxycycline **(B)** by the MAPt analysis. MICs were determined, as described in the *Materials and Methods* section, by the threshold ΔCT = 3.3.

*Bacillus anthracis* is a fast-growing bacterium, and thus, a method that will allow MIC determination directly from patients’ blood is beneficial. A range of *B. anthracis* concentrations were inoculated into human whole blood, which was shaken at 37°C for 1–2 h, yielding a range of bacterial concentrations. The samples were applied to MAPt evaluation. MIC values obtained by MAPt after 7 h of incubation are presented in Table 5 and agree with the MIC values obtained by the standard AST within 2 days (ciprofloxacin 0.008–0.032 μg/ml and doxycycline 0.008–0.032 μg/ml). Graphic representations of the ΔCT results for whole blood are illustrated in Figure 3.

**TABLE 5:** MIC values obtained by MAPt of whole blood samples inoculated with *B. anthracis*.

**Sample (cfu/ml)**	MIC (μg/ml) by MAPt*	
	**Ciprofloxacin**	**Doxycycline**
2.4 × 10^2^	0.008	0.016
3.7 × 10^2^	0.016	0.016
6 × 10^2^	0.008	0.032
3.7 × 10^3^	0.008	0.032
4.7 × 10^3^	0.008	0.032
6 × 10^3^	0.008	0.032
9 × 10^3^	0.016	0.032
10^5^	0.008	0.032
5 × 10^5^	0.016	0.032
10^6^	0.008	0.032
3 × 10^7^	0.016	0.032
MIC by standard AST**		
5 × 10^5^	0.008–0.032	0.008–0.032

Duration of the test: *MAPt: 7 h. **Standard test: 2 days.

### Resistant Ciprofloxacin and Doxycycline Strains of *Yersinia pestis* Are Detected by MAPt

**FIGURE 4:**
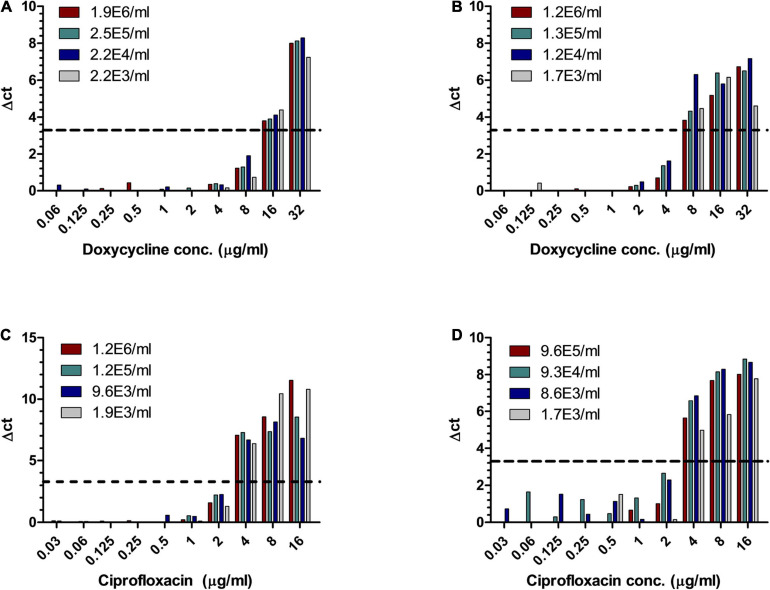
MIC determination of blood cultures and whole blood samples inoculated with *Y. pestis* derivatives with reduced susceptibility to doxycycline and ciprofloxacin. Human blood cultures **(A,C)** and whole blood samples **(B,D)**, inoculated with different concentrations of *Y. pestis* derivatives (cfu/ml) with reduced susceptibility to doxycycline **(A,B)** and ciprofloxacin **(C,D)**, were subjected to AST for ciprofloxacin and doxycycline, by the MAPt analysis. MICs were determined, as described in the *Materials and Methods* section, by the threshold ΔCT = 3.3.

For the proof of concept that MAPt will detect a shift in MIC values as well as a shift in susceptibility categories, we applied MAPt on non-virulent *Y. pestis* derivatives with shifted ciprofloxacin (Steinberger-Levy et al., 2016) and doxycycline (Shifman et al., 2019) MIC values and categories. The same methodology was applied to the resistant strains as to the WT *Y. pestis* strain with extension to antibiotic concentrations that will include the expected elevated MIC values of the resistant derivatives. As can be seen in Table 6 and Figure 4, MAPt can detect elevated MIC values for doxycycline, at various bacterial concentrations, both in blood cultures as well as in whole blood samples, inoculated with a *Y. pestis* derivative with doxycycline-reduced susceptibility. The MIC values obtained were 8–16 μg/ml and were in agreement with the ones detected for this antibiotic-resistant derivative by the standard test; however, the duration of the MAPt was substantially shorter. Importantly, the doxycycline susceptibility breakpoint categories (S—susceptible ≤ 4 μg/ml, I—intermediate = 8 μg/ml, and R—resistant ≥ 16 μg/ml) (Clinical and Laboratory Standards Institute, 2015) were rapidly determined by MAPt. Indeed, the MIC values obtained for the doxycycline derivative alert that doxycycline is not the proper treatment in case of infection with these bacteria.

**TABLE 6:** MIC values for doxycycline obtained by MAPt of blood cultures and whole blood samples inoculated with *Y. pestis* derivatives with reduced susceptibility to doxycycline.

**Sample (cfu/ml)**	MIC (μg/ml) by MAPt*
	**Doxycycline**
**Blood cultures**	
1.8 × 10^3^	16
2.2 × 10^3^	16
10^4^	16
2.2 × 10^4^	16
1.3 × 10^5^	16
2.5 × 10^5^	16
1.5 × 10^6^	16
1.9 × 10^6^	16
**Whole blood**	
10^3^	8
1.7 × 10^3^	8
8 × 10^3^	8
1.2 × 10^4^	8
9.3 × 10^4^	8
1.3 × 10^5^	8
8.6 × 10^5^	8
1.2 × 10^6^	8
MIC by standard AST**	
5 × 10^5^	16–32

Duration of the test: *MAPt: 13 h. **Standard test: 2–3 days.

Similarly, MAPt detected elevated MIC values for ciprofloxacin (Table 7 and Figure 4), at various bacterial concentrations, both in blood cultures and in whole blood samples, inoculated with a *Y. pestis* derivative with ciprofloxacin-reduced susceptibility. The MIC value (4 μg/ml) was in agreement with the ones detected for this antibiotic resistant derivative by the standard test, and define the tested derivative as a non-sensitive bacteria (non-sensitive MIC > 0.25 μg/ml) (Clinical and Laboratory Standards Institute, 2015).

**TABLE 7:** MIC values for doxycycline obtained by MAPt of blood cultures and whole blood samples inoculated with *Y. pestis* derivatives with reduced susceptibility to ciprofloxacin.

**Sample (cfu/ml)**	MIC (μg/ml) by MAPt*
	**Ciprofloxacin**
**Blood cultures**	
1.4 × 10^3^	4
1.9 × 10^3^	4
9.6 × 10^3^	4
1.2 × 10^4^	4
1.2 × 10^5^	4
1.7 × 10^5^	4
1.2 × 10^6^	4
1.9 × 10^6^	4
**Whole blood**	
1.7 × 10^3^	4
2 × 103	4
8.6 × 10^3^	4
1.4 × 10^4^	4
9.3 × 10^4^	4
1.7 × 10^5^	4
9.6 × 10^5^	4
1.9 × 10^6^	4
MIC by standard AST**	
5 × 10^5^	2–4

Duration of the test: *MAPt: 13 h. **Standard test: 2–3 days.

The ability to detect resistant bacteria within a relevant clinical time frame may be a matter of life and death especially in a *Y. pestis* infection where the *in vivo* growth rate of the bacteria is fast compared to the slow growth rate *in vitro* as reflected in the test duration of the standard AST.

## Discussion

A critical barrier to handling BSI and antibiotic resistance is the lack of rapid diagnostics. Consequently, there is improper use of broad first-line antibiotics or a long delay in determining an appropriate antibiotic treatment (Inoue, 2019). The classical gold-standard bacterial antibiotic susceptibility testing, using either a broth microdilution or a disk diffusion assay, requires at least 1 day. Moreover, due to the prior need for an enrichment step in blood culture, following isolation/purification and quantification steps by agar platting, the time to answer can exceed a few days. Ongoing efforts are made to develop improved selective media to advance this path (Aftalion et al., 2021).

Novel rapid methods developed in recent years based on imaging, such as FDA-approved Accelerate Pheno^TM^ (Humphries and Di Martino, 2019), microfluidic cultures, or molecular assays that target resistance genes or proteins (van Belkum et al., 2020), may provide shorter ASTs; however, these novel ASTs cannot be directly applied to whole blood and still require enrichment and isolation steps, maintaining the overall prolonged time to result. Moreover, the presence or absence of a resistance gene does not always correlate with phenotypic resistance or susceptibility (Spencer et al., 2020; Urmi et al., 2020). Efforts to reduce the time of bacteria isolation from blood cultures by plasma purification and immunomagnetic separation, leading to accelerate antibiotic susceptibility determination, have also been reported (Aloni-Grinstein et al., 2017).

MAPt is a rapid and simple AST that circumvents the prerequisite of blood culturing, isolation/purification, and quantification steps demanded by other approaches. The assay is based on direct growth of the bacteria on solid agar embedded with different concentrations of the tested antibiotic. Rapid bacterial growth monitoring, dramatically reducing the time to answer, is achieved by qPCR using specific primers that override potential contaminations that may accompany blood sampling. The use of agar medium, which better supports the growth of bacteria at low concentrations, together with the use of qPCR, which provides sensitivity, allows quantification of relatively low amounts of bacteria (∼5 × 10^2^ cfu/ml). As such, MAPt can be performed directly from blood samples or in cases where less than few hundreds of bacteria are present in 1 ml of blood, after a very short incubation of the blood cultures, allowing time to answer within clinically relevant time frames. Moreover, special attention is needed for Tier-1 bioterror pathogens as beyond prompt treatment of infected patients, and authorities should offer prophylactic management to potentially infected civilians.

Minimal inhibitory concentration values for fast-growing bacteria, such as *B. anthracis*, can be obtained within a few hours (7 h for *B. anthracis*) instead of 1 to 2 days, and for slow-growing bacteria, such as *F. tularensis*, within 16 h, compared to 3–5 days by other means. A challenging clinical situation may occur with *in vivo* fast-growing but *in vitro* slow-growing bacteria such as *Y. pestis*. On one hand, as an *in vivo* fast-growing bacteria, infection with *Y. pestis* may cause deterioration in the patient’s wellbeing within 24 h post symptoms’ onset. However, *Y. pestis* is a slow-growing bacteria *in vitro*; thus, ASTs do not always meet clinical relevance. We show that MAPt can serve as an adequate AST and meet the timewise requirements for establishing targeted antibiotic treatment. *Y. pestis* derivatives, with reduced susceptibility to either ciprofloxacin or doxycycline, were detected within clinical relevance.

In conclusion, MAPt is a novel and rapid AST that can be applied directly to bacteria-infected whole blood samples, with no need for prior procedures such as enrichment in blood cultures, isolation/purification, and quantification steps, thus offering a significantly shorter time-to-answer option with clinical relevance.

## Data Availability Statement

The raw data supporting the conclusions of this article will be made available by the authors, without undue reservation.

## Author Contributions

RA-G, OS, and SR conceived the study. RA-G and SR wrote the manuscript. All authors contributed to the experiments and approved the submitted version.

## Conflict of Interest

Patent application (IL270342) for the described antibiotic susceptibility test (MAPt) was filed by the Israel Institute for Biological Research. The authors declare that the research was conducted in the absence of any commercial or financial relationship that could be construed as a potential conflict of interest.

## References

[B1] Aftalion M. Aloni-Grinstein R. Andrianaivoarimanana V. Lantoniaina Iharisoa A. Shmaya S. Gur D. (2021). Improved selective BIN agar for a better rate of Yersinia pestis isolation from primary clinical specimens in suspected Madagascar plague cases. *J. Clin. Microbiol.* 59:JCM00564. 10.1128/JCM.00564-21 33980652 PMC8288266

[B2] Aloni-Grinstein R. Schuster O. Yitzhaki S. Aftalion M. Maoz S. Steinberger-Levy I. (2017). Isolation of francisella tularensis and yersinia pestis from blood cultures by plasma purification and immunomagnetic separation accelerates antibiotic susceptibility determination. *Front. Microbiol.* 8:312. 10.3389/fmicb.2017.00312 28293231 PMC5329073

[B3] Aloni-Grinstein R. Shifman O. Gur D. Aftalion M. Rotem S. (2020). MAPt: a rapid antibiotic susceptibility testing for bacteria in environmental samples as a means for bioterror preparedness. *Front. Microbiol.* 11:592194. 10.3389/fmicb.2020.592194 33224128 PMC7674193

[B4] Athamna A. Athamna M. Abu-Rashed N. Medlej B. Bast D. J. Rubinstein E. (2004). Selection of *Bacillus anthracis* isolates resistant to antibiotics. *J. Antimicrob. Chemother.* 54 424–428. 10.1093/jac/dkh258 15205405

[B5] Ben-Gurion R. Shafferman A. (1981). Essential virulence determinants of different Yersinia species are carried on a common plasmid. *Plasmid* 5 183–187. 10.1016/0147-619x(81)90019-6 7243971

[B6] CDC (2018). *Tularemia Diagnosis and Treatment.* Available online at: https://www.cdc.gov/tularemia/diagnosistreatment/index.html (accessed February, 2021)

[B7] Clinical and Laboratory Standards Institute (2010). *Methods for Antimicrobial Dilution and Disk Susceptibility Testing of Infrequently Isolated or Fastidious Bacteria: Approved Guidelines-2nd Edn. CLSI Document M45-A2.* Wayne, PA: Clinical and Laboratory Standards Institute.

[B8] Clinical and Laboratory Standards Institute (2015). *Methods for Antimicrobial Dilution and Disk Susceptibility Testing of Infrequently Isolated or fastidious Bacteria 3rd ed. CLSI Document M45-A2.* Wayne, PA: Clinical and Laboratory Standards Institute. 10.1086/510431 17173232

[B9] CLSI (2018). *Methods for Dilution Antimicrobial Susceptibility Tests for Bacteria That Grow Aerobically, CLSI Document M07-11th.* Wayne, PA: Clinical and Laboratory Standards Institute.

[B10] Ellis J. Oyston P. C. F. Green M. Titball R. W. (2002). Tularemia. *Clin. Microbiol. Rev.* 15 631–646. 12364373 10.1128/CMR.15.4.631-646.2002 PMC126859

[B11] Frean J. Klugman K. P. Arntzen L. Bukofzer S. (2003). Susceptibility of Yersinia pestis to novel and conventional antimicrobial agents. *J. Antimicrob. Chemother.* 52 294–296. 10.1093/jac/dkg363 12865386

[B12] Galimand M. Carniel E. Courvalin P. (2006). Resistance of Yersinia pestis to antimicrobial agents. *Antimicrob. Agents Chemother.* 50 3233–3236. 10.1128/aac.00306-06 17005799 PMC1610074

[B13] Goto M. Al-Hasan M. N. (2013). Overall burden of bloodstream infection and nosocomial bloodstream infection in North America and Europe. *Clin. Microbiol. Infect.* 19 501–509. 10.1111/1469-0691.12195 23473333

[B14] Gratz N. Poland J. D. Tikhomirov E. (1999). *Plague Manual: Epidemiology, Distribution, Surveillance and Control.* Available online at: https://www.who.int/csr/resources/publications/plague/whocdscsredc992a.pdf?ua=1 (accessed February, 2021) 10635759

[B15] Guiyoule A. Gerbaud G. Buchrieser C. Galimand M. Rahalison L. Chanteau S. (2001). Transferable plasmid-mediated resistance to streptomycin in a clinical isolate of Yersinia pestis. *Emerg. Infect. Dis.* 7 43–48. 10.3201/eid0701.010106 11266293 PMC2631670

[B16] Heine H. S. Hershfield J. Marchand C. Miller L. Halasohoris S. Purcell B. K. (2015). In vitro antibiotic susceptibilities of Yersinia pestis determined by broth microdilution following CLSI methods. *Antimicrob. Agents Chemother.* 59 1919–1921. 10.1128/aac.04548-14 25583720 PMC4356840

[B17] Hernandez E. Girardet M. Ramisse F. Vidal D. Cavallo J.-D. (2003). Antibiotic susceptibilities of 94 isolates of Yersinia pestis to 24 antimicrobial agents. *J. Antimicrob. Chemother.* 52 1029–1031. 10.1093/jac/dkg484 14613959

[B18] Humphries R. Di Martino T. (2019). Effective implementation of the accelerate pheno system for positive blood cultures. *J. Antimicrob. Chemother.* 74(Suppl 1) i40–i43. 30690541 10.1093/jac/dky534 PMC6382030

[B19] Inglesby T. V. Dennis D. T. Henderson D. A. Bartlett J. G. Ascher M. S. Eitzen E. (2000). Plague as a biological weapon:medical and public health management. *JAMA* 283 2281–2290. 10807389 10.1001/jama.283.17.2281

[B20] Inoue H. (2019). Strategic approach for combating antimicrobial resistance (AMR). *Glob. Health Med.* 1 61–64. 10.35772/ghm.2019.01026 33330756 PMC7731180

[B21] Levy H. Glinert I. Weiss S. Bar-David E. Sittner A. Schlomovitz J. (2014). The central nervous system as target of *Bacillus anthracis* toxin independent virulence in rabbits and guinea pigs. *Plos One* 9:e112319. 10.1371/journal.pone.0112319 25375158 PMC4223028

[B22] Shifman O. Aminov T. Aftalion M. Gur D. Cohen H. Bar-David E. (2021). Evaluation of the european committee on antimicrobial susceptibility testing guidelines for rapid antimicrobial susceptibility testing of *Bacillus anthracis*-, *Yersinia pestis*- and *Francisella tularensis*-positive blood cultures. *Microorganisms* 9:1055. 10.3390/microorganisms9051055 34068310 PMC8153291

[B23] Shifman O. Steinberger-Levy I. Aloni-Grinstein R. Gur D. Aftalion M. Ron I. (2019). A rapid antimicrobial susceptibility test for determining yersinia pestis susceptibility to doxycycline by RT-PCR quantification of RNA markers. *Front. Microbiol.* 10:754. 10.3389/fmicb.2019.00754 31040834 PMC6477067

[B24] Spencer D. C. Paton T. F. Mulroney K. T. Inglis T. J. J. Sutton J. M. Morgan H. (2020). A fast impedance-based antimicrobial susceptibility test. *Nat. Commun.* 11:5328. 33087704 10.1038/s41467-020-18902-x PMC7578651

[B25] Steinberger-Levy I. Shifman O. Zvi A. Ariel N. Beth-Din A. Israeli O. (2016). A rapid molecular test for deternining Yersinia pestis susceptibility to ciprofloxacin by the quantification of differntially expressed marker genes. *Front. Microbiol.* 7:763. 10.3389/fmicb.2016.00763 27242774 PMC4871873

[B26] Sutera V. Hoarau G. Renesto P. Caspar Y. Maurin M. (2017). In vitro and in vivo evaluation of fluoroquinolone resistance associated with DNA gyrase mutations in *Francisella tularensis*, including in tularaemia patients with treatment failure. *Int. J. Antimicrob. Agents* 50 377–383. 10.1016/j.ijantimicag.2017.03.022 28689870

[B27] Tularemia Epidemiology. (2021). *Tularemia Epidemiology.* Available online at: https://www.azdhs.gov/documents/preparedness/epidemiology-disease-control/investigation-manual/vectorborne/tularemia-protocol.pdf (accessed April 25, 2021).

[B28] Urmi U. L. Nahar S. Rana M. Sultana F. Jahan N. Hossain B. (2020). Genotypic to phenotypic resistance discrepancies identified involving beta-lactamase genes, blaKPC, blaIMP, blaNDM-1, and blaVIM in uropathogenic *Klebsiella pneumoniae*. *Infect. Drug Resist.* 13 2863–2875. 10.2147/idr.s262493 32903880 PMC7445497

[B29] van Belkum A. Burnham C. D. Rossen J. W. A. Mallard F. Rochas O. Dunne W. M. Jr. (2020). Innovative and rapid antimicrobial susceptibility testing systems. *Nat. Rev. Microbiol.* 18 299–311. 32055026 10.1038/s41579-020-0327-x

[B30] Versage J. L. Severin D. D. M. Chu M. C. Petersen J. M. (2003). Development of a multitarget real-time TaqMan PCR assay for enhanced detection of *Francisella tularensis* in complex specimens. *J. Clin. Microbiol.* 41 5492–5499. 10.1128/jcm.41.12.5492-5499.2003 14662930 PMC309004

[B31] Weigel L. M. Sue D. Michel P. A. Kitchel B. Pillai S. P. (2010). A rapid antimicrobial susceptibility test for *Bacillus anthracis*. *Antimicrob. Agents Chemother.* 54 2793–2800. 10.1128/aac.00247-10 20439614 PMC2897299

[B32] World Health Organization (2008). *Anthrax in Humans and Animals*, 4th Edn. Available online at: https://www.who.int/csr/resources/publications/anthrax_webs.pdf (accessed April 25, 2021). 26269867

